# Mechanism of crosstalk between DNA methylation and histone acetylation and related advances in diagnosis and treatment of premature ovarian failure

**DOI:** 10.1080/15592294.2025.2528563

**Published:** 2025-07-07

**Authors:** Jing Li, Qianhui Liao, Yurou Guo, Jiaheng Zhang, Ruyi Zhang, Qiyu Liu, Huiping Liu

**Affiliations:** School of Integrated Traditional Chinese and Western Medicine, Hunan University of Chinese Medicine, Changsha, Hunan, China

**Keywords:** Premature ovarian failure (POF), epigenetic regulation, DNA methylation, histone acetylation, non-coding RNA

## Abstract

Premature ovarian failure (POF) affects 1–3.5% of women under 40 years of age, characterized by irreversible depletion of the follicular pool and decline in oocyte quality, with its pathogenesis remaining incompletely understood. Current mainstream therapies, such as hormone replacement therapy, only alleviate symptoms, fail to reverse the underlying functional decline, and carry long-term risks, necessitating the exploration of novel strategies targeting the etiology. This review systematically dissects the central role of epigenetic regulation in POF. First, DNA methylation governs female reproductive lifespan by reprogramming the dormant-activation balance of primordial follicles and maintaining epigenetic memory in oocytes. Second, histone modification homeostasis determines ovarian endocrine function by influencing granulosa cell senescence and steroid hormone synthesis. Additionally, non-coding RNAs form regulatory hubs by constructing competing endogenous RNA networks that integrate oxidative stress and developmental signaling pathways. These mechanisms provide new insights into the pathological basis of POF, identify potential biomarkers, and offer a theoretical framework for deciphering targeted intervention strategies and developing precision epigenetic therapies to delay POF progression.

## Keypolicy highlights

- Promote precision diagnostics using blood-based epigenetic biomarkers like cfDNA methylation and exosomal ncRNAs for early detection.
- Support targeted gene therapies by editing or modulating epigenetic mutations.
- Explore synergistic therapeutic regimens integrating epigenetic drugs with hormone therapy and antioxidants.
- Leverage advanced technologies like single-cell multi-omics and organoid models to predict environment-epigenetic interactions and guide interventions.

## Introduction

The ovary, as the core gonadal organ, houses follicles – the non-renewable functional units whose dormant-activation balance directly determines female fertility [1]. Follicular development is a result of multi-factor regulation from intrinsic to extrinsic cues, where the accumulation of pathogenic genetic information and epigenetic disorders collectively play pivotal roles [2]. Epigenetic regulation, one of the core mechanisms governing premature ovarian failure (POF), primarily encompasses DNA methylation, histone modification, and non-coding RNA (ncRNA) regulation [3]. Among these, DNA methylation – a key epigenetic modification – involves reversible addition/removal of methyl groups at the 5’ carbon position of cytosine in CpG islands, catalysed by DNA methyltransferases (DNMTs) and demethylases (TET). Without altering the DNA sequence, this process regulates gene expression by modifying DNA methylation levels, thereby inducing heritable changes [4,5]. In primordial follicles, DNA methylation levels are relatively low but gradually increase during folliculogenesis, with de novo establishment of specific DNA methylomes occurring during the transition from primary oocytes to secondary follicles [6]. Furthermore, DNA methylation-related molecular biomarkers can serve as indicators for assessing reproductive aging [7]. Histone acetylation, as a dynamic epigenetic modification, also plays a critical role by regulating chromatin conformation and transcriptional processes, and is actively involved in mammalian oocyte maturation [8]. Histone acetylation-modifying enzymes are essential for primordial follicle activation, follicular development, and the maintenance of fertility [9]. However, persistent histone acetylation may lead to reduced gene transcription and ovulatory defects [10]. Thus, histone acetylation not only functions as a key regulator in maintaining reproductive aging homeostasis but also represents a promising therapeutic target for age-related reproductive disorders [11,12]. Furthermore, non-coding (nc)RNAs exert pivotal roles in follicular activation, ovarian function maintenance, and decline by modulating the expression of key folliculogenesis-related genes, redox homeostasis, and the integration of sex hormone-signaling axes [13]. Given the urgent need to extend female reproductive lifespan in an aging society, the current first-line therapy, Hormone Replacement Therapy (HRT), has significant limitations, This review systematically elucidates how epigenetic reprogramming influences ovarian aging through the regulation of follicular development dynamics (activation-atresia balance), reproductive endocrine homeostasis, and oocyte quality maintenance, thereby providing novel perspectives for developing targeted intervention strategies.

## Premature ovarian failure: an urgent frontier in ovarian health research

POF is a disorder characterized by premature decline of ovarian function before the age of 40, marked by reduced primordial follicles, declining oocyte quality, and abnormal female hormone secretion [14]. Common symptoms include menstrual abnormalities, perimenopausal syndrome, and reduced or even lost fertility [15]. Additionally, patients with POF are prone to multiple complications, significantly affecting their quality of life [16]. Despite extensive research, the etiology and pathological mechanisms of POF remain unclear. The reported non-iatrogenic prevalence has increased from 1% in earlier studies to 3.5% in recent data, with a shift toward earlier onset. This trend highlights its characterization as a complex, multifactorial process [17]. POF may be associated with genomic inheritance [18,19], immune system disorders [20], external environmental factors, and lifestyle disruptions [21]. Currently, hormone replacement therapy (HRT) serves as the primary first-line treatment, supplementing hormones to act on target organs and mimic the physiological cycle. However, it fails to effectively restore ovarian function, with the condition prone to recurrence after discontinuation. Long-term use may increase the risk of breast, endometrial, and ovarian cancers [22]. Given the complexity and irreversibility of POF, exploring its pathogenesis and treatment strategies holds significant applied value and scientific significance.

## Association between DNA methylation and POF

### DNA methylation in folliculogenesis and meiosis

Genomic methylation undergoes two reprogramming stages during primordial germ cell (PGCs) formation and early embryonic implantation, involving dynamic reconstruction of global demethylation and de novo methylation [23]. During the window of PGC formation, DNA methylation levels are relatively high. By the sixth week of embryogenesis, PGCs enter meiosis via mitosis and arrest in the prophase diplotene stage, ultimately differentiating into primordial follicles with a hypomethylated state. During follicular development in adolescence (particularly during the transition from primordial follicles [FGOs] to secondary follicles), a specific DNA methylation pattern is reconstructed. In the late stage of follicular development, CpG islands undergo widespread methylation, and FGOs exhibit a bimodal cluster methylation distribution [24]. During late oogenesis, germinal vesicle (GV)-stage oocytes undergo chromatin remodelling, transitioning from the non-surrounded nucleolus (NSN) to the surrounded nucleolus (SN) configuration. This structural reorganization is coupled with dynamic DNA methylation reprogramming mediated by Tet enzymes. These epigenetic modifications not only regulate transcriptional silencing in GV oocytes but also establish specific methylation patterns critical for early embryonic developmental competence [25]. Using an ovarian holding (OH) stress model, our study demonstrated that moderate OH stress (37°C, 1 h) enhances normal NSN-to-SN transition. This process is accompanied by optimized histone modifications and improved oocyte competence, indicating that oocyte functionality is directly linked to the integrity of epigenetic reprogramming. Notably, the mere acquisition of SN morphology does not guarantee developmental potential [26]. Notably, this precise regulatory network is disrupted during aging, characterized by aberrant hypermethylation in primordial follicles and impaired methylation establishment at the FGO stage. These epigenetic aberrations lead to dysregulated follicular activation and diminished oocyte quality. Age-related alterations in leukocyte telomere length and blood methylome profiles possibly correlate with reproductive function. These systemic biomarkers may help delineate phenotypic signatures in females experiencing reproductive decline, potentially serving as quantifiable indicators of reproductive aging [27,28].

### DNA methylation modifiers in oocyte development

DNA methylation, as a crucial epigenetic modification, participates in regulating gene expression networks through its dynamic establishment and precise control. This process is primarily coordinated by DNMTs and TET family proteins, which constitute a ‘molecular switch’ for DNA methylation homeostasis by catalysing the addition and removal of methyl groups, respectively [29]. In mammals, DNMT family members (including DNMT1, DNMT3A, DNMT3B, and DNMT3L) collectively regulate DNA methylation patterns during development through division of labour. DNMT1 is primarily responsible for maintaining existing methylation marks, while DNMT3A and DNMT3B mediate de novo methylation processes [30]. Recent studies have revealed dynamic expression patterns of these methylation-modifying enzymes during oocyte development. Researchers, such as Fatma Uysal, systematically compared the spatiotemporal expression profiles of DNMT family members in oocytes (GV and MII stages) and early embryos across different mammalian species (mouse, cow, and human). The expression levels and subcellular localization of these enzymes exhibit significant species specificity, developmental stage dependency, and are influenced by factors such as in vitro culture conditions and maternal aging [31]. Significant transcriptomic differences and cellular interactions have been noted between oocytes and granulosa cells (GCs) during follicular development in mice. Notably, maternal DNA methylation- or H3K27me3-imprinted genes exhibit cell-type-specific expression patterns, being actively expressed in GCs but silenced in oocytes. This phenomenon suggests that DNA methylation-related enzymes may participate in follicular development regulation by establishing cell-specific epigenetic marks [32]. Zhou’s research [33] further elucidates the molecular mechanism of DNMT1 in granulosa cells. DNMT1 indirectly regulates the expression level of aromatase CYP19A1 by maintaining the methylation status of the long-noncoding (lnc)RNA *IFFD* promoter region, thereby inhibiting granulosa cell apoptosis, influencing oestrogen secretion, and ultimately regulating follicular development. Under conditions involving aging and oxidative stress, Dnmt1 promotes methylation of the Sp1 binding site in the *LEDGF* gene promoter CpG island, suppressing the expression of *LEDGF* and its target Hsp27, leading to reactive oxygen species (ROS) amplification and cellular damage. These findings elucidate its role in aging via the regulation of ROS [34]. These findings provide new insights into the regulatory network of DNA methylation-modifying enzymes in female germ cell development.

### Regulatory factors that specifically bind to methylated DNA

Cullin-RING Ubiquitin Ligase 4 (CRL4) plays a critical role in maintaining oocyte viability and regulating meiotic progression. Yu et al. [35] demonstrated that the E3 ligase DCAF13 of CRL4 regulates oocyte meiosis and maturation by modulating DNA, serving as a key determinant of female fertility. CRL4-DCAF13 interacts with the histone methyltransferase SUV39H1; polyubiquitination and proteasomal degradation of the CRL4 complex lead to rapid oocyte loss, subsequent POF, and silencing of genes essential for fertility maintenance [36]. Damage-specific DNA-binding protein 1 (DDB1), as the adaptor protein of CRL4, acts in concert with the CRL4-associated factor VPRBP (a substrate adaptor of CRL4). VPRBP regulates DNA methylation, sustains oocyte survival, and promotes genomic reprogramming. The complex formed by these components is vital for oocyte development; oocyte-specific deletion of DDB1 or VPRBP alters epigenetic landscapes and delays meiotic resumption [37]. Methyl-CpG-binding protein 2 (MeCP2), an epigenetic regulator that specifically binds to methylated DNA, causes transcriptional dysregulation, DNA hypermethylation, and genomic instability upon overexpression in growing oocytes, ultimately leading to follicular growth arrest and apoptosis. Zhang et al. [38] found that MeCP2 is abundantly expressed in primordial and primary follicles but undetectable in secondary follicles. Compared with young ovaries, the levels of MeCP2 in oocytes and GCs of aged ovaries demonstrate a significant increase. MeCP2 was identified as a substrate of DCAF13 for recognition and targeted degradation via analyses including reduced representation bisulphite sequencing and RNA sequencing. This mechanism prevents DNA hypermethylation in growing oocytes and maintains normal transcription, indicating that the DCAF13-MeCP2 axis is involved in the regulation of ovarian function. The association between DNA methylation regulators (e.g., DNMTs, TETs) and POF pathogenesis is summarized in Table 1, highlighting key regulatory genes and their functional roles.

**Table 1.** The relationships between DNA methylation regulators and POF.

DNA Methylation Regulators	The Relationship with POF	Ref.
Dnmt1	Maintain DNA methylation patterns; participate in oocyte maturation; associate with oocyte apoptosis and autophagy.	[39–41]
Dnmt3A	Participate in de novo DNA methylation; engage in oocyte maturation; elevated expression may inhibit cell apoptosis.	[39,40,42]
Dnmt3B	Participate in de novo DNA methylation.	[39]
Dnmt3L	Differential Expression Patterns and Subcellular Localization in GV vs. MII Oocytes.	[43]
TET	Dysregulation of activity is associated with various ovarian diseases; reduced expression may inhibit oocyte apoptosis.	[44,45]
UHRF1	Methylation levels in oocytes are significantly reduced in UHRF1 knockout mice.	[46]
DCAF13	Participate in DNA methylation-mediated regulation of oocyte meiosis and maturation.	[35]
DDB1	Forming a complex with CRL4-associated factor VPRBP is critical for oocyte development.	[37]
CRL4-DCAF13	Interact with the histone methyltransferase SUV39H1 to maintain oocyte viability.	[36]
MeCP2	Prevent DNA hypermethylation and maintain normal transcription in growing oocytes.	[38]
Stella	Ensure low methylation levels in the genome of mature oocytes.	[47]

### DNA methylation is involved in the pathogenesis of POF

The hypothalamus, pituitary, and gonads form the HPG axis through hierarchical regulation. The core function of this neuroendocrine system is to integrate neural input signals and coordinate reproductive development and homeostasis via pulsatile hormone release (e.g., GnRH, FSH/LH, sex steroid hormones) [48]. Aberrant DNA methylation of key genes in this axis (e.g., *sf-1, cyp19a1a, GnRH*) – such as promoter hypermethylation induced by arsenic exposure or hypoxia – can disrupt sex hormone synthesis (e.g., reduced E2 and 11-ketotestosterone [11-KT] levels) through epigenetic silencing. This disrupts the positive and negative feedback loops of the HPG axis, ultimately leading to POF [49,50]. Additionally, environmental pollutant exposure-induced alterations in DNA methylation patterns play a critical role in the pathogenesis of POF. Female rodents exposed to non-toxic concentrations of uranium for nine months exhibited significant changes in ovarian DNMT1 and DNMT3a/b expression, along with global DNA hypomethylation [51]. Exposure to lead or herbicides disrupted the activity and expression patterns of DNMTs in zebrafish [52–54]. In mice exposed to varying concentrations of cadmium, MeRIP-qPCR and microarray analyses revealed a close association between DNA methylation and the differentiation of embryonic stem cells into ovarian GCs [55,56]. Collectively, these animal studies demonstrate that changes in DNA methylation levels drive ovarian functional abnormalities. Clinical research has also confirmed the link between DNA methylation and disease: DNA methylation regulates gene activation or silencing, and alterations in its expression or patterns increase genomic instability, thereby predisposing to disease [57]. Somayeh et al. [21] found that women’s use of plastic containers alters methylation signatures in cumulus cell genes and proteins, with follicular fluid bisphenol A (BPA) concentrations correlating with poor ovarian response. With patient informed consent, GC samples were collected from a reproductive medicine center during in vitro fertilization or intracytoplasmic sperm injection (IVF-ICSI) treatments. Downregulation of the demethylase FTO increases m6A levels in senescent GCs, while FTO-knockout GCs exhibit faster senescence-associated phenotypes. Hence, FTO may serve as a potential therapeutic target for ovarian aging by acting as an m6A-mediated senescence-delaying protein [58,59]. In summary, environmental, dietary, and lifestyle factors influence DNA methylation, which may, in turn, induce cellular, tissue, and organ aging.

### DNA methylation is associated with stem cell-mediated ovarian function repair

The characteristic pathological changes of ovarian aging include tissue structure atrophy, loss of germ cell regenerative capacity, and functional degradation, making targeted DNA methylation regulation a new strategy. Stem cell therapy, with its multipotent differentiation potential and paracrine effects, has demonstrated the ability to repair ovarian structure, restore hormonal balance, and promote follicle development in animal models, emerging as a research hotspot in regenerative medicine [60,61]. Bone marrow mesenchymal stem cells (BM-MSCs) and hematopoietic stem cells (HSCs), with their multipotent differentiation potential and ability to secrete various cytokines, hold promise as novel therapeutic tools for reversing POF [62]. Multiple studies have confirmed the therapeutic effects of stem cells on POF and their association with DNA methylation. Derany et al. [63] found that epigenetic regulation of signaling pathways, such as TGF-β, Wnt/β-catenin, and Hippo in BM-MSCs, modulates follicular apoptosis and proliferation, contributing to therapeutic effects in radiation-induced POF. Kim et al. [64] demonstrated that repeated transplantation of human placenta-derived mesenchymal stem cells (hPD-MSCs) into aged rats improved ovarian function and promoted follicular resuscitation by regulating DNA methylation expression. Tian et al. [65] established a co-culture system of BM-MSCs and aged GC models, revealing a novel mechanism of m6A methylation modification levels in BM-MSCs during the reversal of GC senescence. As a key regulatory layer for stem cell lineage specification, elucidating the role of DNA methylation in the mechanism of stem cell-mediated ovarian repair may enhance the potential of stem cell therapy for ovarian aging [66].

## Association between histone acetylation and POF

### Histone acetylation is crucial factor in follicular development

A close relationship between histone acetylation and follicular development and function has been noted. Enhancers exist in mouse oocytes, which play a key role in the transcriptional regulation of follicular development, and specific histone acetylation modifications are involved in enhancer regulation [67]. Researchers performed mass spectrometry analysis on GC samples from patients with polycystic ovary syndrome (PCOS) and non-PCOS controls. The results showed 410 differentially acetylated modification sites in the PCOS group, of which 265 proteins exhibited increased acetylation levels and 68 had decreased levels. Changes in lysine acetylation of key enzymes in GCs may affect oocyte maturation and metabolic homeostasis in the follicular microenvironment [68]. In oocytes, histone acetylation mainly occurs at lysine residues of H3 and H4 subunits and changes dynamically with meiosis. Treating oocytes with different concentrations of trichostatin A (TSA) can promote H3K9 acetylation levels, thereby weakening the meiotic process of oocytes [69]. Immunofluorescence showed that mouse GV-stage oocytes had high acetylation of H4K12 lysine, which decreased at germinal vesicle breakdown (GVBD) and disappeared at metaphase II (MII) [70]. H4K5ac, H4K8ac, and H4K16ac signals were strongly enriched in the GV stage, and acetylation was retained to varying degrees in metaphase I (MI) and MII oocytes. Kinetic studies of histone acetylation at specific sites, such as H3K9, H4K12, H4K5, H4K8, and H4K16, have shown that oocyte development is accompanied by changes in acetylation levels, and histone modifications are key epigenetic regulators of oocyte growth [71].

### Histone acetylation-modifying enzymes are involved in the process of oocyte development

Histone acetylation and deacetylation play critical and mutually balancing roles in oocyte development. Histone acetylation promotes the formation of loose chromatin structures, facilitating gene expression and providing a material basis for early oocyte development, whereas histone deacetylation is essential for chromatin condensation and the normal progression of oocyte meiosis. This dynamic balance is jointly regulated by histone acetyltransferases (HATs) and histone deacetylases (HDACs) [72]. HATs include three subfamilies: GNAT, MYST, and p300/CBP. Lysine acetyltransferase 8 (KAT8), highly expressed in mouse GV oocytes, was the first HAT confirmed to significantly regulate follicular development in mice. Overexpression of KAT8 leads to abnormal mitochondrial distribution in oocytes, excessive production of ROS, and subsequent mitochondrial dynamic defects, redox imbalance, and spindle/chromosome disorders [73]. MYST4 protein accumulates in the nucleus of GV oocytes, whereas the p300 and CBP subfamilies are constitutively expressed in a gonadotropin-independent manner during follicular growth and ovulation, regulating follicular development by influencing hyaluronic acid synthesis and cumulus expansion [74].

HDACs are responsible for catalysing the deacetylation of histone lysine residues, categorized into 4 classes and 18 subtypes. Most subtypes are highly expressed in primordial follicles, regulating follicular activation, quiescence, development, and atresia. HDAC1 is expressed in both GCs and oocytes of growing follicles. Sustained expression regulates histone acetylation, influences gene transcriptional activity, and is critical for follicular growth and maturation [75]. HDAC2 maintains relatively stable expression during the follicular growth phase. In pre-ovulatory follicles, its phosphorylation activation synchronizes with histone deacetylation. Silencing or inhibiting HDAC2 leads to reduced gene transcription and ovulation defects [76]. HDAC3 participates in gonadotropin-mediated regulation of oocyte meiosis in granulosa cells. Before the LH surge, FOXO1 recruits HDAC3 to the Areg promoter region to inhibit oocyte maturation; after the LH surge, HDAC3 levels decrease, allowing oocytes to resume meiosis and ovulate [77]. HDAC8 is widely distributed in the cytoplasm of GV-stage oocytes and accumulates around chromosomes after GVBD. Inhibiting its activity causes spindle defects and chromosome misalignment during oocyte meiotic maturation. Panobinostat (PB), an HDAC inhibitor (HDACi) used in clinical cancer treatment, alters histone markers and reduces the number of follicles at all stages in mouse ovaries when administered at 5 mg/kg every other day [78]. The selective HDAC3 inhibitor RGFP966 suppresses HDAC3 activity, leading to hyperacetylation of α-tubulin during oocyte meiosis, which hinders cumulus cell expansion and oocyte meiotic progression [79]. HDAC6 forms complexes with TSC2, TGF-β, NGF, and BDNF, playing a critical role in the transition between primordial follicle quiescence and activation. High HDAC6 expression reduces NGF levels, increases the reserve of primordial follicles in mice, delays their activation, and extends reproductive lifespan, holding promise as a drug target for regulating primordial follicle in vitro activation [80]. Sirtuins (SIRT), NAD±dependent silent regulatory proteins, are crucial for maintaining reproductive aging homeostasis, with SIRT1, SIRT2, SIRT3, and SIRT6 promoting oocyte maturation [81]. HDAC11, the smallest member of the HDAC family, shows gradually decreased expression from the GV to MII stages in oocytes, and inhibiting its activity slows the oocyte maturation rate [82]. In summary, the synergistic action of HATs and HDACs establishes histone acetylation as a key component of the epigenetic modification network in mammalian oocytes. Histone acetylation regulators, including HATs and HDACs, demonstrate significant correlations with POF progression, as detailed in Table 2.

**Table 2.** The relationship between histone acetylation regulators and POF.

Histone acetylation regulators	The relationship with POF	Ref.
HDAC1	It is expressed in both granulosa cells and oocytes of growing follicles, supporting follicular growth and maturation.	[75]
HDAC2	Reduced gene transcription and ovulation defects occur when it is silenced or inhibited.	[76]
HDAC3	It mediates the regulation of gonadotropins on the meiotic process of oocytes.	[77]
HDAC8	It participates in the polar positioning of the spindle during oocyte meiosis.	[78]
HDAC6	It controls the transition between the quiescent and activated states of primordial follicles.	[80]
HDAC11	Inhibiting HDAC11 activity reduces the maturation rate of oocytes.	[82]
Sirtuins (SIRTs)	Improves the capacity for oocyte maturation; participates in ovarian reserve, granulosa cell (GC) function, and oocyte maturation, etc.	[81,83]
SIRT1	p53-SIRT1 mediates or activates the SIRT1/AKT signaling pathway to exert protective activity on damaged granulosa cells (GCs).	[84–86]
SIRT2	It is involved in regulating spindle assembly during oocyte meiosis.	[87]
SIRT3	It alleviates oxidative stress and enhances mitochondrial function in ovarian granulosa cells.	[88]
SIRT5	It provides a basis for the treatment of POI through the protein expression of FOXO3a.	[89]
SIRT6	Reducing the level of H3K9AC alleviates POF and reduces oxidative stress.	[90]
HATs	It regulates follicular development by influencing hyaluronic acid synthesis and cumulus expansion.	[74]
KAT8	Overexpression induces mitochondrial dynamics defects, redox imbalance, and spindle/chromosome disorders in oocytes.	[73]

### Histone acetylation is involved in the onset of POF and treatment

In terms of the pathogenesis of POF, the impact of environmental factors on POF cannot be overlooked. Taking hexavalent chromium (Cr (VI)) as an example, studies from Sivakumar’s laboratory have shown that prenatal exposure to Cr (VI) leads to premature ovarian failure through an acetylation-p53-mediated pathway. In experiments with adult sows, normal drinking water and water containing Cr (VI) were administered during days 9.5–14.5 of pregnancy. Exposure to Cr (VI) in F1 offspring ovaries increased the apoptosis rate of germ cells and reduced the co-localization of acetyl-p53-SIRT1. These findings reveal a novel mechanism by which Cr (VI)-induced ovarian cell apoptosis is mediated by the p53-SIRT1 network [84] and underscore the importance of environmental factors affecting POF through histone acetylation. However, current research on environmental factors is relatively limited, and comprehensive investigations into the combined effects of diet, lifestyle, and other factors on histone acetylation in POF are needed in the future.

**Figure 1.**
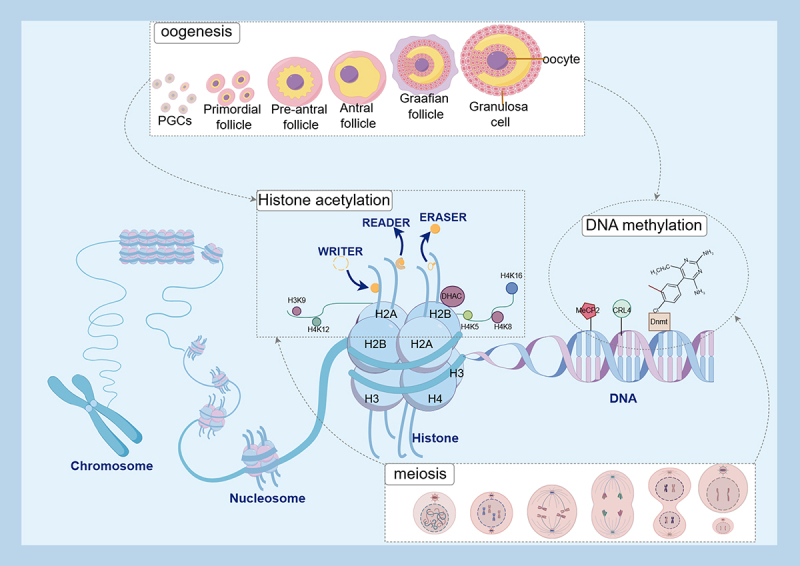
DNA methylation and histone acetylation regulation of follicle development and meiosis.

In the therapeutic research for POF, studies on female germline stem cells (FGSCs) have elucidated novel perspectives. Treatment of FGSCs with metformin modulates their activity via the mitogen-activated protein kinase (MAPK) signaling pathway, with tumour necrosis factor receptor-associated factor 2 (Traf2) identified as a key target of histone 3 lysine 27 acetylation (H3K27ac) during FGSC proliferation. These findings suggest that metformin may exert therapeutic effects on POF through histone acetylation. Moreover, they highlight the potential for other pharmacological agents to influence POF progression via similar epigenetic mechanisms, although current research in this area remains limited [91]. Additionally, multiple substances have demonstrated potential therapeutic value. He et al. [85] found that compound J2 significantly improves the survival rate of GCs damaged by cisplatin, improves cell morphology, reduces apoptosis, and increases oestradiol (E2) levels. J2 exerts its effects by activating the SIRT1/AKT signaling pathway. Given that SIRT1 is characterized as an HDAC, J2 influences histone acetylation. Similarly, Chen et al. [86] found that Danggui Shaoyao San (DSS) upregulates SIRT1 expression and downregulates p53 expression, exerting anti-apoptotic, anti-oxidative stress, and ovarian protective effects. Moreover, SIRT6 reduces H3K9AC and p66SHC levels and weakens p66SHC promoter activity, and overexpression of p66SHC helps alleviate POF symptoms and reduce oxidative stress [90]. Kuntai Capsule (KTC) provides an experimental basis for the treatment of POI by regulating the protein expression of SIRT5 and FOXO3a [89]. These studies indicate that the close association between the SIRT family and histone acetylation through autophagy and oxidative stress is worthy of in-depth exploration. In summary, DNA methylation and histone acetylation regulate follicle formation, development, and meiosis processes (Figure 1).

## DNA methylation and histone acetylation synergistically influence germ cells

### Interaction between DNMTs and HDACs

DNMTs and HDACs jointly regulate gene expression and demonstrate a bidirectional regulatory relationship. The transcriptional repression domain of Dnmt1 can recruit the HDACs, which indicates a more direct connection between DNA methylation and histone acetylation. The DNA methylation process mediated by DNMT1 May alter the chromatin state through the activity of HDACs [92]. Both DNMT1 and DNMT3a can bind to the histone methyltransferase SUV39H1, which restricts gene expression by methylating H3K9. The non-catalytic amino-terminal of DNMT1 binds to DNMT1-associated protein (DMAP1) and HDAC2 to jointly mediate transcriptional repression. During the S phase of DNA synthesis, DMAP1 interacts with the distal N-terminal of DNMT1 to bind to replication foci in a targeted manner, and HDAC2 joins in the late S phase to provide a platform for histone deacetylation in post-replication heterochromatin [93]. Both DNMT3A and DNMT3L contain an ATRX-DNMT3-DNMT3L (ADD) domain, which can recognize the unmethylated histone H3 tail lysine-4 (H3K4me0). Mutations in the DNMT3A-ADD or DNMT3L-ADD domain in germ cells will reduce the global CG methylation level to varying degrees [94].

### Association between DNMTs with H3K4

Histone H3K4 is typically present in promoter regions, and H3K4me3 near it can prevent DNMT3A and DNMT3B from binding to DNA, thereby ensuring that the gene promoter region is not methylated. The gene is typically silenced upon promoter methylation. Abnormalities in H3K4 can increase the DNA methylation level in oocytes, which is an important factor leading to the abnormal developmental potential of oocytes [95]. In vivo experiments using chromatin immunoprecipitation analysis have shown that after the LH surge during ovulation, the levels of histone H4 acetylation (Ac-H4) and H3K4me3 in the steroidogenic acute regulatory (StAR) promoter increase, whereas the levels of H3K9me3 and H3K27me3 decrease; in the aromatase (Cyp19a1) promoter, the levels of Ac-H3, Ac-H4, and H3K4me3 decrease, whereas the level of H3K27me3 increases. These results confirm that DNA methylation and histone modifications jointly influence the expression of *StAR* and *Cyp19a1* and are involved in regulating the chromatin structure of GC promoters during luteinization in ovulation in rats [96].

### Synergistic regulation of Stella, UHRF1 with H3K9 and HDAC1

In 2018, a team of Chinese scientists discovered that DNA methylation regulates the gene *Stella* (also known as *PCG7* or *DPPA3*). In somatic cells, overexpression of *Stella* leads to the formation of a complex with the DNA methylation regulator UHRF1, interfering with the maintenance of DNA methylation modifications during mitosis. In oocytes, high expression of *Stella* prevents the accumulation of UHRF1 in the nucleus, maintaining a low methylation level in the genome of mature eggs. After knocking out *Stella*, the DNA methylation level in oocytes of female mice increases abnormally, primarily in silent gene regions, which remarkably impairs egg quality and leads to infertility. The unique methylation profile of the maternal genome depends on the stability of unmethylated regions. The region with histone H3K9 dimethylation (H3K9me2) binds to *Stella*, which serves to protect these regions from the active demethylation mediated by TET3. Therefore, *Stella* is crucial in the overall DNA methylation and histone acetylation processes [47]. UHRF1, as a hemimethylated CG-binding protein, plays a critical role in maintaining DNA methylation by recruiting DNMT1 to hemimethylated CG sites. Oocytes from mice with UHRF1 knockout exhibit a significant reduction in methylation levels, highlighting its importance in maintaining oocyte methylation [46]. UHRF1 binds to histone H3K9me2/3 and is regulated by HDAC1. Acetylation of UHRF1 at K490 weakens its binding affinity to hemimethylated DNA. Mutating endogenous UHRF1 with acetylation mimics leads to DNA methylation defects in cells, indicating that precise regulation of UHRF1 acetylation is crucial for maintaining DNA methylation during cell division [97]. Table 3 reveals the distinct patterns of DNA methylation and histone acetylation dynamics across key developmental stages during oocyte maturation.

**Table 3.** Stage-specific dynamics of DNA methylation and histone acetylation during oocyte maturation.

Stage	Key DNA Methylation/Histone Acetylation Events	Key Regulators	Ref.
GV	1.Chromatin remodeling from the NSN to SN configuration is accompanied by dynamic DNA methylation reprogramming. 2. Dnmt3L exhibits species- and stage-specific expression patterns. 3. Increased DNMT1 and abnormal H4K5ac levels are observed in aged mouse oocytes. 4. Intense enrichment of H4K12ac, H4K5ac, H4K8ac, and H4K16ac signals. 5. High expression of KAT8 (MYST1). 6. Nuclear accumulation of MYST4. 7. Constitutive expression of p300/CBP. 8. Widespread cytoplasmic distribution of HDAC8. 9. HDAC11 expression gradually decreases from the GV to MII stage.	Tet; Dnmt3L; DNMT1; KAT8; MYST4; p300/CBP; HDAC8; HDAC11	[25]; [31]; [70]; [71]; [73]; [74]; [82]; [98]
GVBD	1. During dynamic changes in DNA methylation. 2. The level of H4K12ac decreases significantly. 3. HDAC8 aggregates from the cytoplasm to the perichromosomal region. 4. Inhibition of HDAC8 activity leads to spindle defects and chromosome misalignment.	HDAC8	[70]; [82]
MI	H4K5ac, H4K8ac, and H4K16ac show acetylation retention to varying degrees.	HDACs	[71]
MII	1. Dnmt3L expression (levels/localization exhibit species and stage differences). 2. DNMTs expression shows significant species specificity and developmental stage dependence. 3. H4K12ac disappears. 4. H4K5ac, H4K8ac, and H4K16ac still retain acetylation to varying degrees. 5. HDAC11 expression continues to decrease. 6. Inhibition of HDAC11 activity slows down the maturation rate.	Dnmt3L; DNMTs; HDAC11	[31]; [43]; [70]; [71]; [82]

## Relationship between the pathogenesis of POF and the crosstalk between DNA methylation and histone acetylation

### Crosstalk between DNA methylation and histone acetylation is involved in the pathogenesis of POF

**Figure 2.**
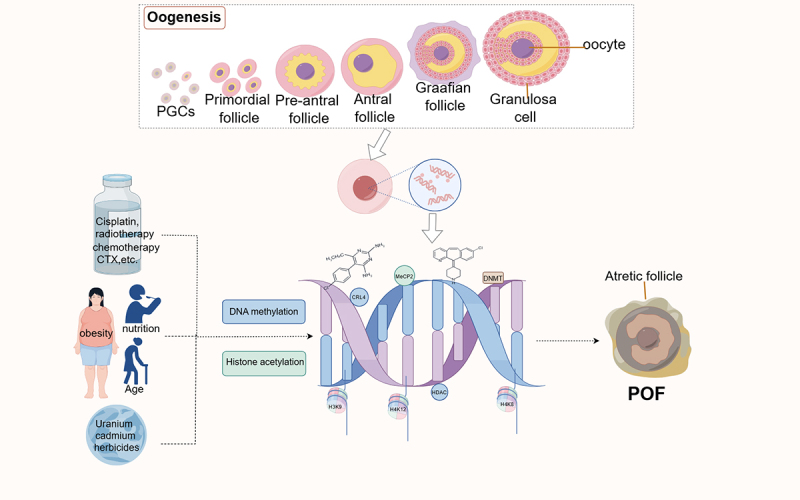
DNA methylation and histone acetylation dysregulation in POF pathogenesis.

Epigenetic mechanisms of environmental exposure and age factors are closely associated with POF. Lactational exposure to Cr (VI) upregulates the expression of DNMT3a/3b and decreases the levels of H3K9ac/H3K27ac in the ovaries of F1 generation mice, leading to follicular apoptosis and developmental disorders [99]. The expression of host defense peptides (HDPs), as important components of the innate immune system, was induced by treating chicken macrophage cell lines with HDACi or DNA methyltransferase inhibitors (DNMTi) alone or in combination, reducing the risk of POF often caused by bacterial and inflammatory ovarian damage. This suggests that bacterial infections may regulate HDP expression through DNA methylation and histone acetylation, thereby influencing ovarian function [100]. In an obese mouse model (high-fat diet-induced obesity), obesity activates DNMTs through the cAMP/PKA/CREB pathway, leading to hypermethylation of oocytes and decreasing SIRT1 expression. Conversely, increasing endogenous SIRT1 expression and reducing p53 levels help reduce follicular apoptosis or atresia and maintain ovarian reserve [101,102]. Studies on GV-stage oocytes from young (10–13 weeks) and aged (69–70 weeks) mice have revealed epigenetic changes in aged oocytes, such as increased DNMT1, decreased MeCP2, and abnormal H4K5ac [98]. These findings indicate that obesity and aging affect follicular development potentially by mediating the interaction between DNA methylation and histone modifications, which jointly regulate gene expression and chromatin structure. Dysregulation of these modifications contributes to the pathogenesis of POF, as demonstrated in Figure 2.

Chemotherapeutic drugs can disrupt the ovarian microenvironment through multiple pathways, including inducing stromal fibrosis, angiogenesis disorders, immune dysfunction, oxidative stress imbalance, and stem cell depletion, thereby significantly damaging follicular quantity and quality. This represents one of the core intrinsic mechanisms mediating chemotherapy-associated ovarian damage (CAOD) and POF [103]. Cisplatin-induced POF is closely linked to abnormal DNA methylation. Treatment with DNMT inhibitor (5-Aza-dC) induces conservative global hypomethylation in BM-MSCs and their secretome, which can significantly restore hormonal levels in damaged ovaries, promote follicular development, and improve structural integrity. This therapeutic effect strongly confirms that abnormal DNA methylation is a key intrinsic mechanism of cisplatin-induced ovarian dysfunction [104]. Cyclophosphamide (CTX) inhibits the activity of histone methyltransferase EZH2, reducing the level of histone H3 lysine 27 trimethylation (H3K27me3), which leads to abnormal activation of pro-apoptotic and transcriptional regulatory genes and induces granulosa cell apoptosis. This finding reveals a new pathway by which chemotherapeutic drugs mediate ovarian damage through interfering with histone methylation [105]. CTX can also reduce the expression of SIRT1, leading to excessive acetylation of the key transcription factor Foxo3a, thereby activating downstream pro-apoptotic pathways (such as PI3K/AKT) and ultimately damaging ovarian reserve function. This indicates that disrupting acetylation homeostasis is an intrinsic mechanism by which CTX mediates POF [106]. In addition, other chemotherapeutic drugs damage the ovaries through mechanisms not dominated by epigenetics. The combined use of cyclophosphamide/busulfan (CY/BUS) induces local and systemic immune disorders in the ovaries and activates the p53/p21/p16 cellular senescence pathway, leading to ovarian structural damage and functional failure [107]. Procarbazine causes follicular oocyte damage and ovarian reserve depletion by directly activating the oocyte apoptosis pathway, inducing local ovarian inflammatory responses, and inhibiting the antioxidant system [108]. Notably, although CY/BUS and procarbazine do not directly focus on DNA methylation or histone acetylation, future studies should explore whether the aforementioned immune disorders, cellular senescence, apoptosis, inflammation, and oxidative stress pathways are regulated by upstream epigenetic modifications to more comprehensively elucidate the complex network of chemotherapy-induced POF. The effects of chemotherapeutic agents on POF, DNA methylation, and histone acetylation are shown in Table 4.

**Table 4.** Epigenetic effects of chemotherapeutic agents inducing POF.

Chemotherapeutic Agent	Primary Pathway to POF	Effect on DNA Methylation	Effect on Histone Acetylation	Ref.
Cisplatin	Disruption of ovarian microenvironment	Induces global/site-specific hypomethylation	Not directly mentioned	[104]
CTX	Disruption of ovarian microenvironment	Not directly mentioned	Inhibits SIRT1 → Foxo3a hyperacetylation; Inhibits EZH2 → H3K27me3 ↓	[105,106]
CY/BUS	Immune dysregulation, activation of p53/p21/p16 senescence pathway	Not directly mentioned	Not directly mentioned	[107]
Procarbazine	Activation of oocyte apoptosis pathways, induction of inflammation, suppression of antioxidant system	Not directly mentioned	Not directly mentioned	[108]

### Intrinsic mechanism underlying crosstalk between DNA methylation and histone acetylation in POF

Researchers used a dihydrotestosterone (DHT)-induced prenatal androgenization (PNA) mouse model and performed methyl-CpG binding domain (MBD) sequencing and methylation-specific polymerase chain reaction (MSP) validation on ovarian samples. The results showed that 857 gene promoter regions in PNA mice were methylated to varying degrees, with downregulated *Dnmt1* expression and global hypomethylation. Furthermore, increased LC3II protein expression and autophagosomes in GCs were observed, accompanied by significantly enhanced mRNA expression of the genes associated with the MAPK/p53 pathway and the autophagy-related gene *Becn1*. This suggests that DHT alters DNA methylation levels, possibly linked to the activation of autophagic processes [41]. As a key regulator of apoptosis, p53 is involved in primordial follicle atresia. SIRT1, SIRT2, and SIRT3 inhibit p53 expression by regulating its acetylation level, reducing follicular apoptosis and atresia to maintain ovarian reserve. Inhibition of T-LAK cell-originated protein kinase suppresses GC apoptosis by regulating the p53/SIRT1 axis, playing a critical role in ovarian follicle development [109]. Studies have confirmed the association between DNA methylation and p53 activity, and the regulation of histone deacetylase SIRT1 also influences p53 expression. Thus, modulating p53 has been hypothesized to impact the expression patterns of DNA methylation and histone acetylation, thereby regulating follicular growth and development. Abnormal genomic methylation in embryonic stem cells (ESCs) hinders the differentiation and therapeutic application of their derivatives. Research has found that Sirt1 protein inhibits Dnmt3L transcription and interacts with Dnmt3L protein to deacetylate and destabilize it. By antagonizing Dnmt3L, Sirt1 selectively prevents abnormal DNA methylation of partial developmental genes in mouse ESCs [110].

Oxidative stress is a critical factor affecting oocyte quality. Gene expression differences primarily stem from DNA methylation. In aged human oocytes, mitochondrial dysfunction associated with GC senescence leads to increased ROS production in mice.Female mouse cells generate more ROS and have fewer antioxidant substances, rendering them more susceptible to oxidative stress damage. Exposure of cells to DNMTi can block methylation-induced gene expression differences [111]. The SIRT family plays a key role in genome maintenance, metabolism, and aging, participating in regulating physiological processes such as ovarian reserve and follicle development [83]. SIRT2 is primarily located in the cytoplasm and regulates spindle assembly and chromosome segregation during oocyte meiosis. A low acetylation level of spindle assembly checkpoint protein 1 (BubR1) reduces the incidence of spindle assembly errors and chromosome segregation abnormalities in oocytes, improving egg quality [87]. SIRT3 is mainly localized in mitochondria and serves as an important mitochondrial deacetylase; its deficiency accelerates ovarian aging. The SIRT pathway can mitigate oxidative stress damage, enhance mitochondrial function, protect against cisplatin-induced apoptosis of ovarian granulosa cells, maintain ovarian peroxide levels, promote follicle development, and delay ovarian aging [88]. Therefore, oxidative stress may be one of the mechanisms through which the crosstalk between DNA methylation and histone acetylation regulates follicular growth and development.

## Exploring new mechanisms for diagnosis and treatment of POF based on the crosstalk between DNA methylation and histone acetylation

### Improving POF through environmental signals, such as diet

**Figure 3.**
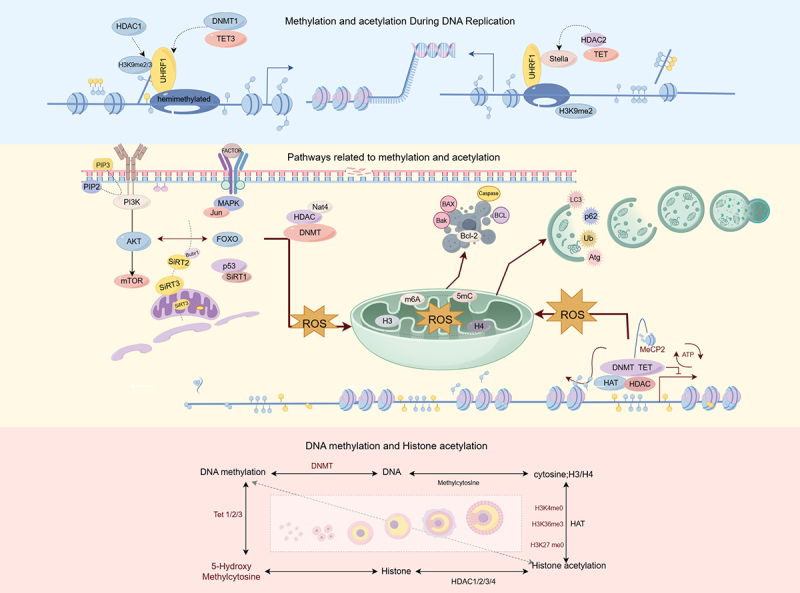
Crosstalk mechanism between DNA methylation and histone acetylation in POF.

Caloric restriction (CR), a strategy that reduces caloric intake while ensuring essential nutrient intake, has been studied for its effects on DNA methylation markers of aging. CR can alter aging-related molecules and slow down the aging process [112]. With age, DNA methylation patterns drift, characterized by increased or decreased methylation levels at different loci. In rhesus monkeys subjected to CR, age-related methylation drift was significantly mitigated, resulting in a blood methylation age approximately seven years younger than their chronological age [113]. The histone *N*-α-terminal acetyltransferase NAT4 regulates cellular lifespan. NAT4 deficiency prolongs the replicative lifespan of yeast, and the longevity attributed to NAT4 activity exhibits penetrance with the effects of CR; its expression weakens CR-mediated lifespan extension [114]. These findings indicate that CR is closely linked to DNA methylation and histone acetylation, participating in aging retardation through environmental signals, such as diet. This provides new insights for the prevention and treatment of POF, suggesting that lifestyle modifications may protect ovarian function. The crosstalk between DNA methylation and histone acetylation drives POF progression through synergistic epigenetic modifications (Figure 3).

### Improving POF through the interaction of DNA methylation, histone modifications, and ncRnas

DNA methylation and histone modifications form an epigenetic-transcriptomic cascade network by regulating the expression and function of ncRNAs, jointly maintaining ovarian granulosa cell homeostasis and follicular development. The TAB2 antisense long non-coding RNA (TAB2-AS) functions with DNA methylation modifications to co-activate antioxidant factors such as SOD1 and P50/P65, inhibit GC oxidative stress and apoptosis, promote proliferation, and accelerate follicular growth [115]. The ‘DNA methylation-TAB2-AS-TAB2’ pathway reveals the dual role of ncRNAs as epigenetic effectors; they are not only regulated by DNA methylation but also enhance target gene stability through RNA-RNA interactions. Histone deacetylases (such as SIRT1/SIRT2) participate in ovarian aging by regulating ncRNA activity. miR-181a promotes FoxO1 acetylation-mediated granulosa cell apoptosis by inhibiting SIRT1, forming an ‘acetylation-miRNA-apoptosis’ positive feedback loop that can interfere with ovarian function [116]. Acetylation at the K512 site of heat shock cognate protein Hsc70 enhances chaperone-mediated autophagy activity, significantly alleviating GC senescence, whereas the activity of its deacetylase SIRT2 can be indirectly regulated by ncRNA, forming an ‘acetylation modification – ncRNA – cellular homeostasis’ regulatory loop [117]. Additionally, metformin activates the MAPK signaling pathway and promotes FGSC proliferation by enhancing histone H3K27 acetylation at the *Traf2* gene locus, demonstrating that small-molecule drugs can reverse ncRNA-mediated stem cell functional defects by remodelling histone modification landscapes [91]. These studies have established an intervention pathway of ‘modifying enzyme regulation → histone/DNA modification → ncRNA network → cellular function.’ In the future, combining single-cell epigenomic sequencing technologies to dynamically monitor the spatiotemporal interaction patterns between ncRNAs and epigenetic modifications is expected to develop precision intervention strategies based on triple regulation of ‘acetylation-methylation-ncRNA,’ demonstrating a novel strategy for clinical translation in POF.

### Cross-regulation of DNA methylation and histone acetylation in assisted reproductive technologies

With the widespread application of assisted reproductive technologies, the impact of epigenetic modifications on embryonic development has become increasingly prominent. Cai et al. [118] summarized the main epigenetic modifications in mammals and their synergistic effects, analysing their regulatory mechanisms from oogenesis to embryonic development. They found that imbalances in nutrition (proteins, lipids, and one-carbon metabolism) can disrupt epigenetic modifications, leading to abnormal oocyte development. By reviewing modifications at key loci and their underlying molecular mechanisms, nutritional status was elucidated to influence oocyte and embryo development during in vitro fertilization, through factors such as DNA methylation and histone acetylation. Oocyte cryopreservation is a critical component of in vitro fertilization-intracytoplasmic sperm injection, but vitrification freezing can influence the epigenetics of oocytes and embryos. Compared with the control group, the vitrification group showed significantly reduced relative transcriptional abundances *of DNMT1, DNMT3B, HDAC1*, and *SUV39H1* in oocytes, as well as significantly decreased DNA methylation levels and H3K9 acetylation levels in two-cell embryos. Hence, oocyte vitrification may interfere with key stages of epigenetic reprogramming during preimplantation embryo development [119]. DNMTs are affected by in vitro culture conditions, and abnormal DNA methylation can lead to increased cell stress and apoptosis, thereby reducing blastocyst quality. In a mouse in vitro culture experiment, after adding baicalin, the expression of Dnmt1 decreased and that of Dnmt3a increased in in vitro-cultured blastocysts, and reducing cell stress and apoptosis and improving embryonic developmental capacity [42]. Additionally, acetylation changes at the histone H4 lysine 12 (acH4K12) site in mouse oocytes influenced fertilization and subsequent embryonic development. Significantly increased AcH4K12 levels in aged oocytes may alter acetylation patterns during fertilization and reduce oocyte developmental potential [120]. Therefore, a deeper understanding of epigenetic regulatory mechanisms in assisted reproductive technologies is of great significance for optimizing technical procedures and improving success rates. The components involved in the cross-talk between DNA methylation and histone acetylation in modulating POF-related pathways are systematically compared in Table 5, elucidating potential synergistic mechanisms.

**Table 5.** Cross-talk of DNA methylation and histone acetylation and POF.

The Crosstalk between DNA Methylation and Histone Acetylation Regulates POF		Ref.
Dnmt1 and HDACs	The transcriptional repression domain of Dnmt1 exerts its function by recruiting the activity of HDACs.	[92]
Dnmt1 and HDAC2	Participates in the S phase of DNA synthesis and jointly mediates transcriptional repression.	[93]
ADD and H3K4me0	The ADD domain binds to the unmethylated histone H3 tail at H3K4me0.	[94]
DNMT3A/B and H3K4	Ensures that the gene promoter region is not methylated by DNA.	[95]
UHRF1 and HDAC1	UHRF1 is regulated by HDAC1, and acetylation of UHRF1 weakens its binding affinity to hemimethylated DNA.	[97]
Stella and H3K9me2	The H3K9me2 region binds to Stella to protect it from active demethylation by TET3.	[47]
DNMT3a/3b and H3K9ac	Increasing DNMT3a/3b and decreasing H3K9ac and H3K27ac disrupt follicular development.	[99]
DNMT1 and H4K5ac	Show synergistic effects in the extrachromatin of aging oocytes.	[98]
DNA methylation and SIRT3 regulate p53	Regulates cell apoptosis and primordial follicles by modulating p53.	[109]
Sirt1 and Dnmt3L	Sirt1 deacetylates Dnmt3L protein and loses its stability	[110]
Oxidative stress in follicles	DNA methylation and histone acetylation cross-regulate the oxidation and antioxidation processes in female germ cells and follicles.	[83,111, 121–123]
DNMT1、DNMT3B、HDAC1 and SUV39H1	The transcriptional abundance in vitrified oocytes is reduced.	[119]

## Summary and prospects

As important components of epigenetics, DNA methylation and histone acetylation are involved in all stages of follicular development from primordial germ cells and are crucial for the establishment of oocyte competence, normal follicular growth, and maturation. Their dynamic changes are finely regulated by DNMTs, TETs, HATs, HDACs, and related factors. Furthermore, DNA methylation and histone modifications interact through diverse mechanisms to jointly regulate gene expression and chromatin states, thereby influencing ovarian function and reproductive health. In the diagnosis and treatment of POF, environmental signals, such as diet (e.g., caloric restriction), stem cell repair, and assisted reproductive technologies are closely related to the regulation of DNA methylation and histone acetylation, providing new strategies, mechanisms, ideas, and research directions for improving ovarian function, treating POF, and enhancing the success rate of assisted reproduction. These mechanisms confirm that epigenetic disorders are the core drivers of POF and may serve as key targets for treating age-related reproductive issues. Regulating the expression of related target genes or modifying enzymes is expected to improve POF, providing a theoretical basis for targeted interventions.

These findings offer promising avenues for further exploration; however, sufficient relevant evidence is needed for clinical applications in the treatment of ovarian aging. Future research should mainly focus on the following aspects: (1) In-depth mechanism analysis: Mapping ovarian-specific epigenetic landscapes, elucidation of the interaction network between DNA methylation clocks and histone modification markers, and dissecting epigenetic regulatory axes, such as their coupling with molecular signaling pathways related to ovarian function. (2) Precision diagnostic applications: Development of blood-based epigenetic markers (e.g., cfDNA methylation, exosomal ncRNA) for early warning of ovarian reserve decline, which could serve as new methods for early diagnosis of ovarian aging. (3) Breakthroughs in targeted therapy: Employing gene editing technologies to target and correct relevant epigenetic mutations (e.g., abnormal FOXO3 methylation); use of high-throughput screening to identify small-molecule compounds or biological agents that regulate epigenetic expression or activity, such as DNMT/HDAC/SIRT modulators; and exploration of synergistic regimens combining epigenetic drugs with traditional therapies (HRT, antioxidants). (4) Integration of technological innovations: Strengthening fundamental research on the physiological functions of epigenetics in the reproductive system and combining emerging technologies, such as single-cell multi-omics and organoid models, to establish a dynamic prediction system for “environment-epigenetics-ovarian function. By dissecting the epigenetic regulatory network from multiple dimensions, precise intervention strategies for extending female reproductive lifespan can be developed.

## Data Availability

No datasets were generated or analysed in this study.
